# Enhancing Timeliness and Compliance of Osteoporosis Care in Oncology: Evidence from a Dedicated Bone Health Service

**DOI:** 10.3390/jcm14186564

**Published:** 2025-09-18

**Authors:** William Balzi, Valentina Danesi, Andrea Roncadori, Ilaria Massa, Roberta Maltoni, Nicola Gentili, Martina Cavallucci, Alice Andalò, Laura Ridolfi, Venetia Zavoiu, Maria Cristina Focherini, Raffaele Giannini, Enrico Campadelli, Stefano Tamberi, Sebastiano Calpona

**Affiliations:** 1 Outcome Research, IRCCS Istituto Romagnolo per lo Studio dei Tumori (IRST) “Dino Amadori”, Via P. Maroncelli 40, 47014 Meldola, Italy; william.balzi@irst.emr.it (W.B.); valentina.danesi@irst.emr.it (V.D.); andrea.roncadori@irst.emr.it (A.R.); ilaria.massa@irst.emr.it (I.M.); roberta.maltoni@irst.emr.it (R.M.); 2 Data Unit, IRCCS Istituto Romagnolo per lo Studio dei Tumori (IRST) “Dino Amadori”, Via P. Maroncelli 40, 47014 Meldola, Italyalice.andalo@irst.emr.it (A.A.); 3 Osteoncology and Rare Tumors Center (CDO-TR), IRCCS Istituto Romagnolo per lo Studio dei Tumori (IRST) “Dino Amadori”, Via P. Maroncelli 40, 47014 Meldola, Italy; laura.ridolfi@irst.emr.it (L.R.); sebastiano.calpona@irst.emr.it (S.C.); 4 Experimental and Clinical Oncology of Immunotherapy and Rare Cancers, IRCCS Istituto Romagnolo per lo Studio dei Tumori (IRST) “Dino Amadori”, Via P. Maroncelli 40, 47014 Meldola, Italy; venetia.zavoiu@irst.emr.it; 5 Internal Medicine and Rheumatology Unit, Infermi Hospital, AUSL Romagna, 47924 Rimini, Italy; mariacristina.focherini@auslromagna.it; 6 Geriatrics Unit, Infermi Hospital, AUSL Romagna, 48018 Faenza, Italy; raffaele.giannini@auslromagna.it; 7 Medical Oncology, Santa Maria delle Croci Hospital, AUSL Romagna, 48121 Ravenna, Italy; enrico.campadelli@auslromagna.it (E.C.); stefano.tamberi@auslromagna.it (S.T.)

**Keywords:** breast neoplasms, retrospective studies, real-world evidence, bone density conservation agents, patient care management, clinical pathway, postmenopausal osteoporosis

## Abstract

**Background/Objectives**: Management of cancer treatment-induced bone loss (CTIBL) is essential for preserving quality of life among breast cancer (BC) patients receiving endocrine therapy. However, bone-modifying agents (BMAs) remain underused and delayed. In 2014, IRST launched the first bone health outpatient service in Romagna (the eastern area of the Emilia-Romagna region). A multi-centre, retrospective observational study with propensity score matching (PSM) was conducted to evaluate the impact of the IRST organisational model on bone health. **Methods**: The PSM matched the Emilia-Romagna patients who underwent BC surgery between 2014 and 2022 and were in follow-up in the Romagna area. Patients were grouped as follows: (1) IRST and (2) other Romagna hospitals (without bone health service, i.e., the control group). The matching was based on age, in situ/invasive cancer, and type of early-stage treatment (hormone treatment vs. chemotherapy). Logistic regression and Cox proportional-hazard models assessed factors associated with bone care treatment initiation and timings, respectively. **Results**: After PSM, we matched 3112 of the 8021 eligible patients into the two cohorts. IRST patients were 39% more likely to receive BMAs (OR: 1.393; 95% CI: 1.236–1.571) and initiated treatment approximately 12 months earlier. We observed that patients with invasive tumours were 77% more likely to initiate bone therapy than those with in situ tumours (OR: 1.766; 95% CI: 1.237–2.585). The early initiation of bone health therapy was influenced by age (*p* < 0.001) and neoadjuvant chemotherapy treatment (*p* < 0.001). **Conclusions**: The IRST model demonstrates responsiveness to bone health needs in BC patients and may be implemented elsewhere to support integrated CTIBL care.

## 1. Introduction

Cancer and its treatment can significantly affect bone health, increasing the risk of skeletal complications [1,2,3,4]. Due to the high survival rates and prolonged cancer treatment, early-stage BC patients represent a key at-risk group for bone loss [3]. Most anticancer treatments, particularly endocrine therapies, accelerate bone resorption and impair bone formation, leading to a condition known as cancer therapy-induced bone loss (CTIBL), which negatively impacts the patient’s quality of life [2,5]. CTIBL is responsible for reduced bone mineral density (BMD), which results in patients’ osteopenia and osteoporosis [2]. These conditions increase the risk of fragility fractures, which are associated with morbidity and poor prognosis [6]. Women treated for BC may encounter up to a 70% increased incidence of osteoporosis [7], a 35% increased risk of fracture [8], and a 25% increased risk of hospitalization due to fracture [6]. Thus, the early identification of cancer patients at high risk for CTIBL is crucial for the optimal management of early-stage BC to preserve bone health. International and national guidelines recommend a proactive approach to managing CTIBL, which includes a baseline risk assessment, pharmacological intervention, and lifestyle modification [1,9,10,11]. Among the available medications, bone-modifying agents (BMAs), such as bisphosphonates and denosumab, are effective in maintaining bone density and reducing the risk of fractures. In some cases, they can also improve cancer treatment outcomes [12]. Despite guidelines encouraging the use of BMAs, evidence suggests these interventions are often not offered to early-stage BC women at high risk of osteoporosis fracture [13,14,15]. Many studies have shown that bisphosphonates and denosumab are not used enough in high-risk women, with up to one-third not receiving any treatment for bone health during their hormone therapy [16,17,18], leading to much higher fracture rates compared to those who were treated. A survey of Canadian oncologists further corroborates the issue of under-treatment, as some physicians did not offer bisphosphonates to all eligible patients, applying personal criteria more restrictive than guidelines [19]. This gap in treatment is caused by several reasons, such as worries about side effects, difficulties with logistics (like needing to visit often for drug infusions), not following guidelines properly, not being recognized as a fracture risk by doctors and patients, and confusion about which healthcare professionals should handle this care.

Moreover, randomized clinical trials have demonstrated that delaying the initiation of bone health therapy in patients with early-stage breast cancer (BC) receiving endocrine treatment is associated with clinically significant skeletal deterioration [20,21]. On the other hand, starting bisphosphonate or denosumab therapy early, at the same time as adjuvant endocrine treatment, has been proven to effectively stop CTIBL and lower the rates of clinical fractures. In the ABCSG-18 trial, the addition of denosumab at treatment onset halved the incidence of first clinical fractures compared to placebo (HR: 0.50, *p* < 0.0001), with sustained benefit over a 7-year follow-up [22]. Similarly, immediate initiation of zoledronic acid in postmenopausal women led to increases in BMD and was associated with improved disease-free survival compared to delayed treatment [20]. These findings support the early implementation of antiresorptive therapy to preserve bone health and potentially enhance oncologic outcomes.

Several studies suggest that structured pathways improve care delivery in oncology and osteoporosis. These approaches include structured clinical pathways, multidisciplinary teams, and dedicated bone health outpatient services integrated into oncology care. At Gemelli Hospital in Rome, the introduction of a new diagnostic and therapeutic assistance pathway, featuring early specialist referral and a dedicated case manager, significantly increased the rates of Dual-Energy X-ray screening and the timely initiation of antiresorptive therapy [23]. A structured bone health program in Jordan improved BMD in up to 84% of high-risk patients [24]. In Canada, an educational nurse-led initiative raised patient awareness from 40% to 96%, thereby improving adherence to preventive strategies [25].

Since 2014, the IRCCS Istituto Romagnolo per lo Studio dei Tumori “Dino Amadori” (IRST) has established an outpatient specialised bone health service integrated into the oncology pathway of BC patients [26]. In particular, the project started in 2014, focusing on BC women, and later, it was extended to various types of cancer, such as prostate, brain cancers, and haematological tumours. The IRST’s bone specialist plays a crucial role in diagnosing, treating, and managing osteoporosis and other metabolic bone diseases, providing rehabilitation and prevention measures to improve patients’ bone health. All IRST patients treated pharmacologically or undergoing surgery that may affect bone health are referred to the IRST outpatient specialized service in healthy bone for a baseline evaluation of bone status, including Dual-Energy X-ray Absorptiometry (DXA) of the lumbar spine and the hip and a panel of bone metabolism blood tests, followed by the prescription of antiresorptive treatment, if appropriate. Patients undergo annual follow-up with a serum bone metabolism profile and hip and spine DXA every two years until the end of endocrine therapy. A final follow-up is scheduled at the end of the AI treatment. This initiative was not entirely unprecedented in the Romagna area. In 2013, the Local Health Authority (AUSL Romagna) introduced a similar pathway at the Faenza hospital, specifically within the Osteoporosis Outpatient Clinic and the Oncology Unit. However, that service was limited to a single hospital of AUSL Romagna, involving a limited number of patients, in contrast to the broader, large-scale model later developed at IRST. Notably, both IRST and AUSL Romagna operate within the same geographical territory and serve the same patient population with comparable epidemiological and disease characteristics, thereby minimising any potential confounding by demographic or clinical differences.

The primary objective of this analysis is to evaluate the impact of the IRST organisational model on the timeliness of bone health care in BC compared to the standard care (no bone health service) provided in the hospitals of the Local Health Authority.

## 2. Materials and Methods

A multi-centre, retrospective observational study was conducted to assess the impact of the IRST organisational model on bone health, targeting the population of women with BC. The study population and their data were retrieved retrospectively from the administrative databases of the Emilia-Romagna region, which served as unique sources to identify patient cohorts and data. BC cases were identified based on the International Classification of Diseases, Ninth Revision, Clinical Modification (ICD-9-CM) [27].

### 2.1. Study Population and Case Selections

**Figure 1:**
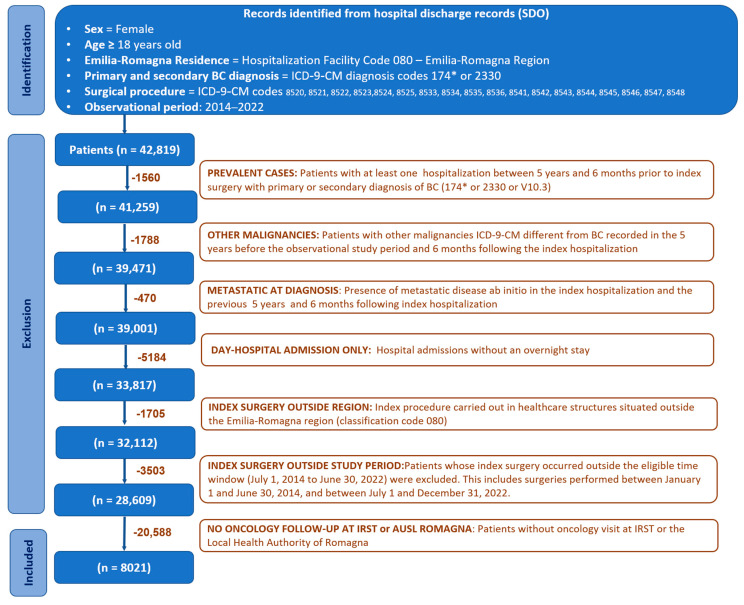
Patient disposition. * 174 (ICD-9-CM) code refers to malignant neoplasm of female breast (all subcategories).

The study included all adult incident female patients residing in the Emilia-Romagna region who were diagnosed with breast cancer and underwent breast surgery at a hospital within the Emilia-Romagna region between July 2014 and June 2022, and who received oncological follow-up within the Romagna area (Figure 1). 

BC case identification started with hospital discharge records (SDO), from which BC surgical procedures were selected based on the following ICD-9-CM codes: 85.41-48 (mastectomies), 85.22 (quadrectomies), 85.23 (subtotal mastectomies), 85.20-21 (removal of breast tissue and lesions), 85.24-85.25 (removal of ectopic breast tissue and nipple), and 85.33-36 (mammectomy). These procedures were considered when performed in any healthcare facility in the Emilia-Romagna region (regional code 080). Eligible patients required a primary or secondary diagnosis of invasive breast cancer, identified by ICD-9-CM codes 174.0-174.9. Additionally, cases with a primary or secondary diagnosis of in situ breast carcinoma (ICD-9-CM code 233.0) were also included. We excluded patients with evidence of metastatic disease from 1825 days (5 years) before to 180 (6 months) days after the index hospitalization, as identified by ICD-9-CM codes 197.0-197.8, 198.0-198.8, and 199.0-199.1. The 5-year presurgical and 6 month postsurgical window was applied to exclude prevalent and undetected metastatic cases, ensuring inclusion of true early-stage breast cancer patients. Furthermore, patients without any recorded oncology visits at IRST or any hospital facility belonging to the Local Health Authority (AUSL Romagna) were excluded from the study. Female patients diagnosed with malignancies other than BC within five years before the start of the study period or in the six months following the index hospitalization were also deemed ineligible. Prevalent cases were excluded by identifying patients who had undergone BC surgery between 1825 and 180 days before the index intervention and who were in oncological follow-up within the Romagna district (i.e., at least one recorded oncology visit).

### 2.2. Cohort Assignment

The eligible population in oncological follow-up within the Romagna district was divided into two groups:

- The patients receiving cancer care at IRST with a dedicated bone health service;
- The control population consisting of patients receiving cancer care at hospitals in the Romagna district that did not have a dedicated bone health service, with the exception of the Faenza hospital.

Cohort assignment was based on the frequency of oncology visits at IRST compared to other hospitals in Romagna during the accrual period. A patient was classified in the IRST cohort if the number of her visits to IRST exceeded those to other hospitals; conversely, the patient was assigned to the control cohort. Patients were followed from the date of BC surgical procedures, defined as the index date, until the end of the observational period (December 2022) or until death, whichever occurred first. Demographic and clinical data were obtained from administrative databases. The initiation date of antiresorptive treatment was defined as the first dispensation of oral drugs or the first infusion of intravenous agents, as recorded in the administrative databases, within a time window ranging from 180 days before the index surgery to the end of the observational period (31 December 2022). The use of a unique patient identification code assigned to all residents of Emilia-Romagna, regardless of the care setting (inpatient or outpatient), enabled deterministic record linkage across the various databases. This study was approved by the Scientific Medical Board of IRST IRCCS and Area Vasta Romagna.

### 2.3. Statistical Analysis

Patients receiving cancer care at IRST or those followed in other facilities (i.e., the IRST vs. control group) were matched 1:1 without replacement using a nearest neighbour propensity score. The propensity scores for receiving cancer care at IRST were estimated using a logistic regression model accounting for age, tumour type (invasive vs. in situ), neoadjuvant hormone therapy, neoadjuvant chemotherapy, and adjuvant treatment (both endocrine treatment and chemotherapy). Furthermore, previous anti-osteoporosis (OP) treatment was also included in the logistic regression model for controlling for potential pre-exposure. Given the nature of the study, the population size was not determined by a statistical power computation; practically, all consecutive patients who met the inclusion and exclusion criteria during the accrual period were included.

Means, standard deviations (SDs), medians, and ranges were used to describe quantitative variables as appropriate. Absolute frequencies, together with proportions, were used to summarize categorical variables. Furthermore, post-matching, absolute risk differences (ARDs), and number needed to treat (NNT) were derived from BMA initiation rates between cohorts to enhance clinical interpretability.

Time-to-event variables (e.g., time to bone health treatment initiation) were summarised using Kaplan–Meier (KM) survival estimates, and differences between groups were formally assessed with the log-rank test. Furthermore, a Cox proportional-hazard (PH) regression model was developed to investigate the factors associated with the timing for treatment initiation. The main analysis considered time to antiresorptive treatment initiation from surgery; however, in the subgroup of patients receiving adjuvant chemotherapy, it was also deemed clinically relevant to evaluate the time to initiation of antiresorptive therapy starting from the beginning of adjuvant treatment.

Additionally, the time to antiresorptive treatment initiation in an adjuvant setting was evaluated using competing risk methodology, considering metastatic progression or death prior to treatment as a single competing event. Cumulative incidence functions (CIFs) were estimated and plotted to visualize differences in the probability of event occurrence. Accordingly, the association between baseline covariates and the risk of treatment initiation (event of interest) or metastatic progression/death (competing event) was formally assessed using the Fine–Gray proportional-subdistribution-hazard model. Coherently, subdistribution hazard ratios (sHRs) with 95% confidence intervals (CIs) and *p*-values were reported.

Lastly, a logistic regression model was used to determine the determinants associated with the likelihood of starting the antiresorptive treatment under analysis.

Missing data were expected to be entirely random (MCAR). All reported *p*-values lower than 0.05 were considered statistically significant, and tests, unless otherwise specified, were two-tailed. All analyses were performed using R Statistical Software version (version 4.4.2, R Foundation for Statistical Computing, Vienna, Austria)

## 3. Results

Based on the search query applied to the regional administrative databases, 8045 female patients were identified. Among these, 24 patients were excluded from the analysis because they had an equal number of medical visits at both IRST and local health authorities, making it impossible to assign them to a specific cohort. The final number of patients analysed consisted of 8021 patients, divided into two groups: the IRST group (N = 3112; 38.8%) and the control group (N = 4909; 61.2%). Table 1 summarises the distribution of demographic and clinical characteristics between the two groups before the application of PSM. The median age of patients in the IRST group was 62 years, compared to 64 years in the control group. In both cohorts, only a small number of patients received neoadjuvant treatments. Specifically, chemotherapy was administered to 5.9% of IRST patients and 4.1% of those in the control group. In comparison, hormone therapy was prescribed to 4.8% of IRST patients and 2.0% of those in the control group. Before PMS, the proportions of patients who received antiresorptive therapy before surgery were well balanced between the two groups, with 3.6% in the IRST cohort and 3.4% in the control group. In both groups, postoperative hormone therapy was the most frequently administered treatment, received by 82.5% of IRST and 73.2% of control group patients, followed by postoperative chemotherapy, administered to 27.9% and 20.6% of patients, respectively.

After applying PSM (see Supplementary Materials Table S1 for details on the multivariable logistic regression model) and verifying the fulfilment of common support requirements (see Supplementary Materials Figure S1), the final matched cohort comprised 6224 patients (N = 3112 for each group). Table 2 summarises the demographic and clinical characteristics of the two groups after matching. Specifically, the distribution of propensity scores for both matched and unmatched individuals in the IRST and control group is illustrated in Supplementary Figure S2. Nearly all *p*-values in the matched population were not statistically significant (*p* > 0.05), indicating a good balance of covariates between the two cohorts. Additionally, after adjustment, covariate balance between the two groups substantially improved. As shown in the love plot (Supplementary Figure S3), all baseline variables achieved absolute standardized mean differences well below the conventional 0.1 threshold, indicating that the adjustment procedure was effective in reducing systematic differences between groups. However, a significantly higher proportion of patients treated at IRST received anti-osteoporosis drugs compared to those in the control group (30.4% vs. 24.8%, *p* < 0.0001). This corresponds to an absolute risk difference of 5.5%, yielding a NNT of 18.1, indicating that for every 18 patients managed under the IRST model, one additional patient received bone-modifying agents compared to the control group. Among the medications dispensed after index surgery, denosumab was used at a lower rate in the IRST cohort (3.9%) compared to the control group (5.2%).

According to the multivariable logistic regression model (Table 3), patients referred to IRST were significantly more likely to initiate bone health therapy compared to those in the control group (OR: 1.393; 95% CI: 1.236–1.571; *p* < 0.0001), corresponding to a 39.3% increased likelihood. As expected, patients who had already received bone health therapy before surgery were much more likely to continue treatment after surgery, with an over 61-fold increase in the odds of initiating postsurgical therapy (OR: 61.557, 95% CI: 37.091–109.490, *p* < 0.0001). Similarly, the use of postsurgical hormone therapy was strongly associated with the initiation of bone health treatment (OR: 4.887, 95% CI: 3.902–6.188, *p* < 0.0001).

Patients with invasive tumours had a +76.6% higher likelihood of initiating bone health therapy than those with in situ tumours (OR: 1.766, 95% CI: 1.237–2.585, *p* = 0.0024). Conversely, age was not statistically significant at the start of treatment (OR: 1.004, 95% CI: 0.999–1.008, *p* = 0.1288), despite its clinical relevance.

**Figure 2:**
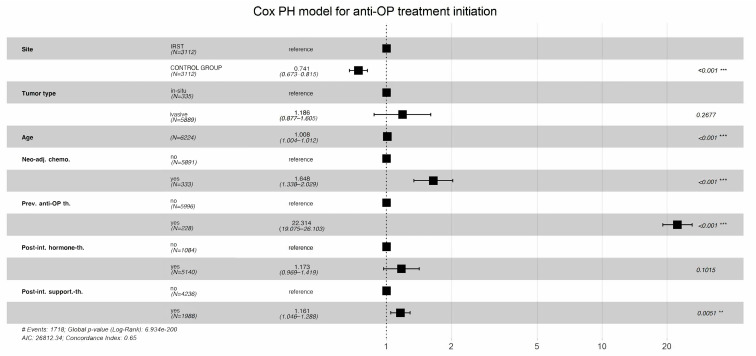
Multivariable Cox proportional-hazards model assessing factors associated with the time to initiation of bone health treatment after surgery. Asterisks indicate levels of statistical significance ** *p* < 0.01, *** *p* < 0.001.

According to the multivariable Cox proportional-hazards regression model, which evaluated factors associated with the time to initiation of bone health treatment, patients followed at IRST experienced a significantly shorter time to treatment initiation compared to those in the control cohort (HR: 0.741, 95% CI: 0.673–0.815, *p* < 0.001), while holding all other covariates in the model fixed (Figure 2). Older age was associated with a modest but statistically significant acceleration in treatment initiation (HR: 1.008 per year, 95% CI: 1.0004–1.012, *p* < 0.001). Additionally, having received neoadjuvant chemotherapy was significantly associated with earlier treatment initiation (HR: 1.648, 95% CI: 1.338–2.029; *p* = 0.001). Conversely, tumour type (HR: 1.186, 95% CI: 0.877–1.605, *p* > 0.2) and receipt of neoadjuvant hormone therapy (HR: 1.173, 95% CI: 0.969–1.419, *p* > 0.1) were not significantly associated with earlier initiation of bone health treatment.

**Figure 3:**
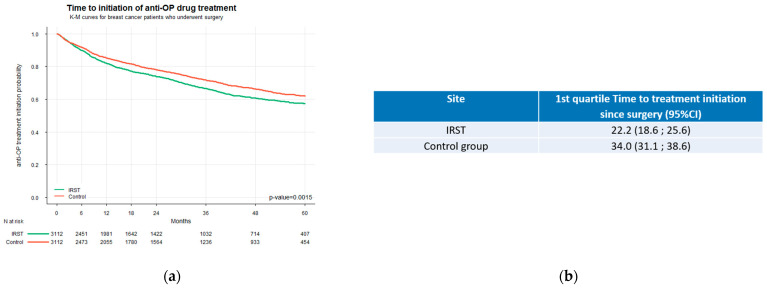
(**a**) Time to initiation of bone health therapy (from breast surgery); (**b**) comparison of the first quartile (25th percentile) time to initiation of antiresorptive therapy.

An analysis based on Kaplan–Meier estimates revealed a clear and statistically significant difference in the timing of bone health treatment initiation between the two care settings (*p* < 0.0001) (Figure 3a). Treatment was initiated approximately one year earlier in IRST patients than in the control group. The first quartile (25th percentile) of IRST patients initiated bone health treatment within 22.2 months from surgery (95% CI: 18.6–25.6 months), whereas patients in the control group reached this threshold one year later at 34.0 months (95% CI: 31.1–38.6 months) (Figure 3b). Moreover, the Kaplan–Meier curves began to diverge immediately after surgery, indicating a greater likelihood of earlier treatment initiation during the early postoperative period for the IRST group. The absence of overlap between the two confidence intervals, since the upper bound of the IRST group (25.6 months) was lower than the lower bound of the control group (31.1 months), confirms a statistically significant delay in treatment initiation among patients in the control group.

**Figure 4:**
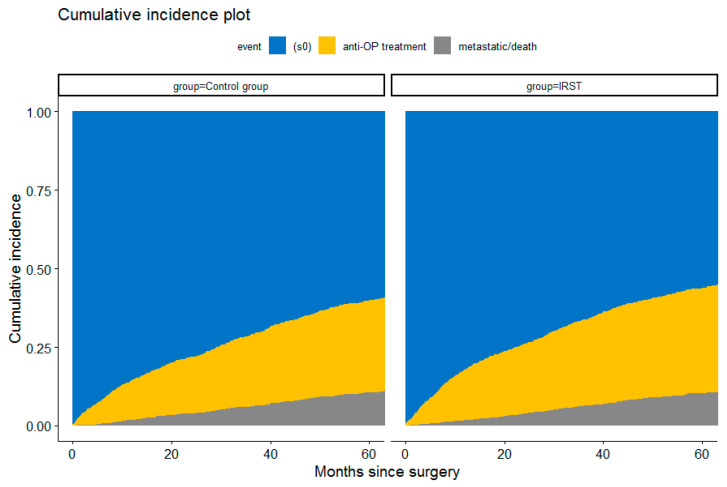
Cumulative incidence plot of adjuvant antiresorptive treatment initiation and competing risk of metastatic progression or death by study group. Stacked cumulative incidence functions are shown for both the control group (**left**) and IRST (**right**) over 60 months following surgery. Yellow areas represent initiation of antiresorptive treatment, grey areas represent metastatic progression or death, and blue areas indicate patients remaining event-free.

In the competing risk analysis, initiation of antiresorptive treatment was more frequent and occurred earlier in patients treated at the CTIBL prevention facility compared with the control group (Figure 4).

Furthermore, as shown in Table 4, the Fine–Gray model confirmed this effect, showing a significantly higher subdistribution hazard of treatment initiation in the facility group (sHR = 1.22, 95% CI 1.10–1.36, *p* = 0.0003). Interestingly, no difference was observed in the risk of metastatic progression or death (sHR = 0.97, 95% CI 0.82–1.14, *p* = 0.67), further supporting the hypothesis of comparable cohorts. Not surprisingly, older age was associated with slightly earlier treatment initiation (sHR = 1.01, 95% CI 1.00–1.01, *p* = 0.003), but also with a substantially increased risk of metastatic progression or death (sHR = 1.06, 95% CI 1.05–1.07, *p* < 0.0001). Patients with invasive tumours did not differ in treatment initiation compared with those with in situ tumours but showed nearly a two-fold higher risk of metastasis or death (sHR = 1.97, 95% CI 1.01–3.87, *p* = 0.048). Conversely, previous exposure to antiresorptive therapy strongly predicted treatment initiation (sHR = 18.51, 95% CI 14.45–23.70, *p* < 0.0001) and was associated with a lower risk of metastatic progression or death (sHR = 0.22, 95% CI 0.11–0.44, *p* < 0.0001). Similarly, postsurgery hormone therapy markedly increased the likelihood of antiresorptive treatment initiation (sHR = 2.77, 95% CI 2.25–3.41, *p* < 0.0001) and reduced the risk of metastasis or death (sHR = 0.46, 95% CI 0.38–0.56, *p* < 0.0001).

**Figure 5:**
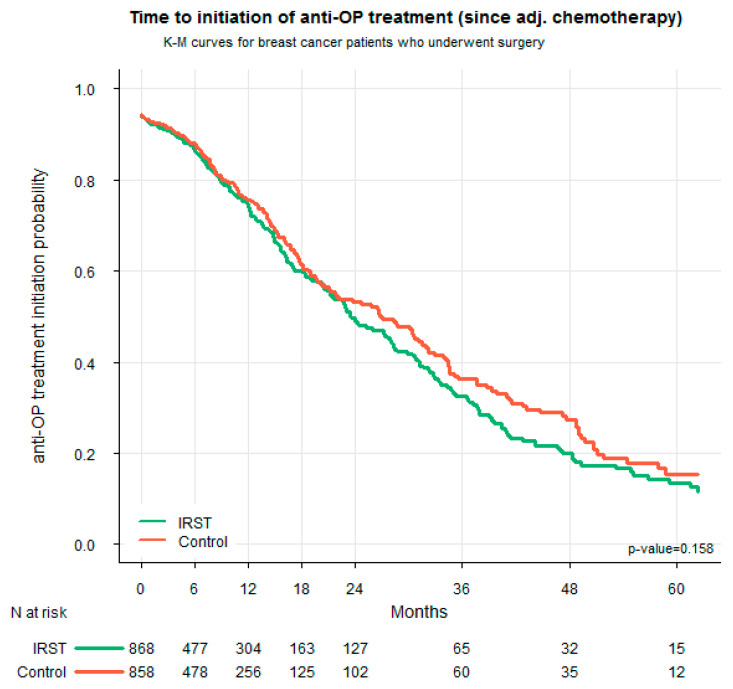
Time to initiation of bone health therapy in the subgroups of patients treated with adjuvant chemotherapy. The initiation time was calculated from the start of adjuvant chemotherapy.

In the subgroup of patients who received adjuvant chemotherapy (IRST: N = 868; Control: N = 858), no significant difference was observed in the timing of the initiation of bone health treatment. The Kaplan–Meier survival curves were largely overlapping, and the log-rank test was not statistically significant (*p* = 0.158), indicating that the time to initiate treatment was comparable between the two groups (Figure 5).

## 4. Discussion

This study provides robust real-world evidence supporting the clinical and organisational value of integrating a dedicated bone health service into the care pathway for patients with early-stage breast cancer. Our findings indicate that women managed at IRST, where a dedicated bone health service has been in place since 2014, were significantly more likely to receive bone-modifying therapy compared to those followed in hospitals without such a service. The marked difference observed in the first quartile of time to treatment initiation underscores the effectiveness of the IRST organisational model in ensuring timely access to bone health therapy. Notably, IRST patients in the 25th percentile initiated treatment within 22.2 months from surgery (95% CI: 18.6–25.6), whereas patients in the control group reached the same percentile nearly one year later, at 34.0 months (95% CI: 31.1–38.6). The non-overlapping confidence intervals between the two groups strongly support the presence of a systematic and clinically relevant difference in early treatment uptake. Consistently, the competing risk analysis further supported these findings, showing that antiresorptive treatment was initiated earlier and more frequently in patients managed at IRST, without differences in the risk of metastatic progression or death, thus reinforcing the comparability of the two cohorts. These results highlight the impact that dedicated services and integrated care models can have in reducing delays in the management of bone health in BC patients. The initiation of treatment occurred approximately one year earlier among IRST patients, a difference that may carry substantial clinical implications in terms of fracture prevention and long-term skeletal preservation. In contrast, the delay in the control cohort may be attributable to a more fragmented care process and the absence of standardized referral procedures for bone health evaluation. The use of denosumab was slightly lower in the IRST cohort compared to the control group (3.9% vs. 5.2%), despite overall higher rates of bone therapy administration. This result likely reflects a deliberate effort to optimize resource allocation based on patient-specific risk profiles. In fact, a local risk stratification score was developed at IRST to support clinicians in selecting the most appropriate therapy according to clinical needs and to guide the optimal use of resources, particularly in the current context of healthcare austerity. This approach is consistent with the Italian regulatory framework (e.g., Nota 79), which defines appropriateness criteria without favouring a specific agent across all subgroups [28]. The tool serves as an additional criterion to assess patient risk profiles across different subgroups, aiming to ensure more targeted and efficient management of bone health interventions. However, in this study, we provided only a snapshot of the bone-targeted treatments without examining the underlying prescription criteria; therefore, we cannot conclude whether IRST or the control group demonstrates better prescribing practices. It is important to mention that the slightly higher use of denosumab in the control group might be partly due to the organised treatment plan at the Faenza centre, where most patients in the special outpatient clinic were specifically given denosumab.

Overall, patients in the IRST group received oncological treatments more often than those in the control group, both in the neoadjuvant and adjuvant settings, consistent with the specialized and comprehensive care approach of the IRST cancer centre. However, the study’s findings indicate that older patients and those who receive neoadjuvant chemotherapy are more likely to initiate bone health therapy earlier, regardless of the institutions where they received care. Another important advantage of the IRST model is its systematic, longitudinal approach, from baseline DXA and metabolic screening at the start of endocrine therapy to annual follow-up visits and final reassessment at the end of treatment. This comprehensive strategy facilitates the early identification of high-risk patients, continuous monitoring of bone status, and timely adaptation of therapy.

Moreover, the literature provides evidence that promoting patients’ education can improve compliance with treatment [29]. As a result of this analysis, patient information leaflets were developed and distributed across all oncology departments of the local health authority of Romagna and IRST. These materials were designed to raise awareness among cancer patients about bone health, improve knowledge, encourage proactive engagement, and promote timely discussions with healthcare professionals about the prevention and management of cancer treatment-induced bone loss.

The positive impact of similar initiatives has been reported internationally. Structured bone health programs in centres such as the Gemelli Hospital in Rome, the King Hussein Cancer Center in Jordan, and various Canadian institutions have led to improved adherence to guidelines, stabilization or improvement of BMD, and greater patient awareness [24,25]. Our findings reinforce the value of such multidisciplinary, integrated approaches and suggest that models like IRST’s could be successfully adopted in other healthcare settings to address the underuse of bone-modifying agents in early breast cancer care.

Moreover, the relevance of a dedicated bone health service is even greater in the current clinical landscape, where adjuvant endocrine therapies are increasingly extended beyond 5 years for 70–80% of early BC patients, often reaching up to 10 years of treatment [2,30]. If the management of CTIBL is still a critical component of supportive care, the prolonged exposure to AIs significantly intensifies the cumulative risk of bone loss and fragility fractures over time. This underscores the urgent need for structured, proactive bone health monitoring and intervention models, such as the one implemented at IRST, to ensure timely prevention, treatment, and follow-up in high-risk populations.

Another critical barrier to the effective management of CTIBL is the limited cultural and scientific attention that this topic still receives within the oncology community. Despite its well-documented impact on morbidity and long-term quality of life, bone health remains a secondary concern in many cancer care settings. This cultural underrepresentation contributes to the insufficient dissemination of information not only among healthcare professionals but also among patients themselves, resulting in low awareness, delayed recognition of symptoms, and underutilisation of preventive strategies. Bridging this knowledge gap through targeted educational initiatives and professional training is essential to enhance both clinician responsiveness and patient engagement.

This study has limitations inherent to retrospective, observational designs. Data were derived from administrative databases, and clinical variables, such as BMD values, actual fracture incidence, specific risk factors, and adherence to therapy, could not be assessed. Although propensity score matching was applied to reduce selection bias, residual confounding cannot be entirely excluded. Furthermore, the study does not evaluate direct clinical outcomes (e.g., fracture reduction or quality of life) associated with earlier or more frequent treatment, which should be explored in future prospective analyses or by combining the current data with modelling approaches to better estimate fracture prevention benefits [31]. It must also be taken into account that many healthcare activities and procedures in this field are carried out outside the National Health System for various reasons, including the availability of Dual X-ray Absorptiometry, heterogeneity in oncologists’ approaches, and the involvement of other medical specialities besides oncology. While this may represent a limitation for data collection, the establishment of the Comprehensive Cancer Care Network in the Romagna region can, on the other hand, help to raise awareness of bone health needs among all stakeholders (patients, healthcare professionals, and physicians) and contribute to improving the management and treatment of this condition. A further limitation of this study is that it was not possible to analytically isolate the specific data from Faenza within the overall AUSL della Romagna dataset, so the control population was analysed as a single cohort without distinguishing the local pathway of Faenza Hospital focused on bone health. However, this local pathway, although structured and active up to the present day, involved relatively small patient numbers that were insufficient to statistically influence the overall results. If any bias had been introduced, it would likely have resulted in an underestimation of the actual impact of the IRST model, as Faenza’s structured pathway was included within the control group.

The present analysis, despite its limitations, demonstrates that the organisation of care can significantly impact outcomes. Including bone health as part of routine oncology care, through dedicated services, early screening, and structured follow-up, can significantly improve access to therapy. Future research should focus on evaluating the long-term clinical outcomes of these interventions, refining patient stratification tools and examining their cost-effectiveness across different healthcare settings.

## 5. Conclusions

These findings underscore the importance of integrating bone health management into oncology care pathways to reduce therapeutic inertia and improve clinical benefits in breast cancer patients at risk of bone complications.

## Figures and Tables

**Table 1:** Baseline demographics and clinical characteristics of patients before propensity score matching. *p*-values are reported for comparisons between the two cohorts.

	Pre-Matching		
Characteristics	IRST Group N = 3112 (%)	Control Group N = 4909 (%)	*p*-Value
Median age—years [IQR ^1^]	62 [52; 72]	64 [53; 73]	<0.0001
In situ breast cancer	188 (6.0)	637 (13.0)	<0.0001
Neoadjuvant chemotherapy	184 (5.9)	201 (4.1)	0.0002
Neoadjuvant hormone therapy	148 (4.8)	99 (2.0)	<0.0001
Neoadjuvant supportive medications ^2^	199 (6.4)	200 (4.1)	<0.0001
Antiresorptive therapy ≤ 180 days presurgery	113 (3.6)	168 (3.4)	0.6201
Adjuvant chemotherapy	868 (27.9)	1026 (20.9)	<0.0001
Adjuvant hormone therapy	2566 (82.5)	3594 (73.2)	<0.0001
Postsurgery supportive medications ^2^	1007 (32.4)	1045 (21.3)	<0.0001

^1^ Interquartile Range. ^2^ Neoadjuvant and postoperative supportive medications include aprepitant, dexamethasone, epoetin alfa, filgrastim, and ondansetron.

**Table 2:** Baseline demographics and clinical characteristics of patients after propensity score matching. *p*-values are reported for comparisons between the two cohorts.

	Post-Matching		
Characteristics	IRST Group N = 3112 (%)	Control Group N = 3112 (%)	*p*-Value
Median age—years [IQR ^1^]	62 [52; 72]	62 [51; 72]	0.120
In situ breast cancer	188 (6.0)	147 (4.7)	0.021
Neoadjuvant chemotherapy	184 (5.9)	149 (4.8)	0.049
Neoadjuvant hormone therapy	148 (4.8)	97 (3.1)	0.0009
Neoadjuvant supportive medications ^2^	199 (6.4)	159 (5.1)	0.029
Antiresorptive therapy ≤ 180 days presurgery (≥1 agent)	113 (3.6)	115 (3.7)	0.893
Bisphosphonates	108 (3.5)	110 (3.5)	0.935
Alendronate	76 (2.4)	77 (2.5)	0.157
Risedronate	21 (0.7)	22 (0.7)	0.878
Ibandronate Sodium	11 (0.4)	7 (0.2)	0.345
Zoledronic acid	2 (0.1)	0 (0.0)	0.157
Neridronate Sodium	0 (0.0)	8 (0.3)	0.008
Clodronic Acid	2 (0.1)	0 (0.0)	1.000
Denosumab	5 (0.2)	5 (0.2)	1.000
Adjuvant chemotherapy	868 (27.9)	858 (27.6)	0.777
Adjuvant hormone therapy	2566 (82.5)	2574 (82.7)	0.789
Postsurgery supportive medications ^2^	1007 (32.4)	981 (31.5)	0.480
Antiresorptive therapy dispensed after surgery (≥1 agent)	945 (30.4)	773 (24.8)	<0.0001
Bisphosphonates	863 (27.7)	670 (21.5)	<0.0001
Alendronate	665 (21.4)	524 (16.8)	<0.0001
Risedronate	170 (5.5)	104 (3.3)	<0.0001
Ibandronate Sodium	38 (1.2)	15 (0.5)	0.002
Zoledronate	32 (1.0)	45 (1.4)	0.136
Neridronate Sodium	9 (0.3)	8 (0.3)	0.808
Clodronic Acid	2 (0.1)	3 (0.9)	0.655
Denosumab	120 (3.9)	161 (5.2)	0.012
Median Time ^3^ to antiresorptive treatment from surgery, in days [IQR ^1^]	NA (927; NA)	NA (1255; NA)	0.0003

^1^ IQR, Interquantile range. ^2^ Neoadjuvant and postoperative supportive medications include aprepitant, dexamethasone, epoetin alfa, filgrastim, and ondansetron. ^3^ Cumulative Incidence Function estimation (Fine and Gray). NA: Not Achieved.

**Table 3:** Multivariable logistic regression model evaluating factors associated with the initiation of bone health treatment. Odds Ratios (ORs), standard error (SE), 95% confidence interval (CI), Z-values, and *p*-values are reported for each covariate included in the model.

Parameter	Odds Ratio (OR)	SE	95% CI		Z	*p*-Value
			Lower Limit	Upper Limit		
(Intercept)	0.026	0.007	0.016	0.042	−14.343	<0.0001
Site (IRST vs. Control group)	1.393	0.085	1.236	1.571	5.431	<0.0001
Tumour type (invasive vs. in situ)	1.766	0.331	1.237	2.585	3.032	0.0024
Age	1.004	0.002	0.999	1.008	1.519	0.1288
Previous anti-OP treatment	61.557	16.900	37.091	109.490	15.007	<0.0001
Postsurgery chemotherapy	1.470	0.147	1.209	1.789	3.853	0.0001
Postsurgery hormone therapy	4.887	0.574	3.902	6.188	13.501	<0.0001
Postsurgery supportive therapy	1.349	0.130	1.116	1.628	3.107	0.0019

**Table 4:** Fine–Gray competing risk regression for time to antiresorptive treatment initiation and for metastatic progression or death. Subdistribution hazard ratios (sHRs) with 95% confidence intervals (CIs) and *p*-values are reported for each covariate.

Fine and Gray Model	Antiresorptive Treatment Initiation				Metastatic Disease/Death			
Parameter	sHR	95% CI		*p*-Value	sHR	95% CI		*p*-Value
		Lower Limit	Upper Limit			Lower Limit	Upper Limit	
CTIBL prevention facility	1.218	1.095	1.355	0.0003	0.965	0.815	1.142	0.670
Age	1.006	1.002	1.010	0.003	1.058	1.048	1.068	<0.0001
Tumour type (invasive vs. in situ)	1.127	0.852	1.491	0.400	1.974	1.005	3.874	0.048
Previous antiresorptive treatment	18.506	14.453	23.698	<0.0001	0.219	0.108	0.444	<0.0001
Postsurgery hormone therapy	2.770	2.249	3.411	<0.0001	0.463	0.382	0.562	<0.0001

## Data Availability

The raw data presented in this study are available at a reasonable request from the corresponding author.

## Supplementary Materials

The following supporting information can be downloaded at https://www.mdpi.com/article/10.3390/jcm14186564/s1: Table S1: Multivariable logistic regression; Figure S1: Common support requirement assessment; Figure S2: Distribution of propensity scores after the nearest neighbour matching; Figure S3: Love plot for covariate balance assessment before and after adjustment.

## Abbreviations

The following abbreviations are used in this manuscript:

BC	Breast Cancer
BMAs	Bone-Modifying Agents
BMD	Bone Mineral Density
CI	Confidential Interval
CIFs	Cumulative Incidence Functions
CTIBL	Cancer Treatment-Induced Bone Loss
DXA	Dual-Energy X-ray Absorptiometry
HR	Hazard Ratio
ICD-9-CM	International Classification of Diseases, Ninth Revision, Clinical Modification
IQR	Interquartile Range
IRCCS	Scientific Institute for Research, Hospitalization and Healthcare
KM	Kaplan–Meier
MCAR	Missing Completely at Random
OP	Osteoporosis
OR	Odds Ratio
*p*	*p*-value
PH	Proportional-Hazard
PSM	Propensity Score Matching
SD	Standard Deviation
SDO	Hospital Discharge Records
SE	Error Standard
sHR	Subdistribution Hazard Ratio
Z	Z-value
