# Supplementary material for: Enhancing Timeliness and Compliance of Osteoporosis Care in Oncology: Evidence from a Dedicated Bone Health Service
Source: J Clin Med. 2025 Sep 18;14(18):6564. doi: 10.3390/jcm14186564 (PMC12470544; doi:10.3390/jcm14186564)
Supplement: Supplementary file 1 [file jcm-14-06564-s001.zip › jcm-3829379-supplementary.pdf]

Table S1. Multivariable logistic regression. The analysis was used to estimate the propensity scores of belonging to the IRST cohort (i.e., receiving cancer care at IRST).

Logit model						
**Parameter**	**Odds Ratio (OR)**	**SE**	**95% CI**		**Z**	**p-value**
			**Lower limit**	**Upper limit**		
(Intercept)	0. 345	0. 050	0. 260	0. 456	-7. 404	<0. 0001
Tumor type (invasive vs. in-situ)	1. 683	0. 163	1. 394	2. 038	5. 375	<0. 0001
Age	0. 994	0. 002	0. 99	0. 998	-3. 129	0. 0018
Neo-adj. chemotherapy	0. 900	0. 185	0. 599	1. 344	-0. 513	0. 6081
Neo-adj. hormone-therapy	2. 213	0. 312	1. 681	2. 924	5. 628	<0. 0001
Previous anti-OP treatment	1. 157	0. 147	0. 900	1. 483	1. 144	0. 2525
Neo-adj. supportive-therapy	1. 434	0. 282	0. 977	2. 116	1. 835	0. 0666
Post-surgery chemotherapy	0. 825	0. 068	0. 702	0. 969	-2. 340	0. 0193
Post-surgery hormone-therapy	1. 532	0. 099	1. 351	1. 739	6. 622	<0. 0001
Post-surg. supportive-therapy	1. 941	0. 152	1. 665	2. 265	8. 463	<0. 0001

Abbreviation: CI, confidential interval; SE Standard error; Z, Z statics; Neo-adj., Neo-adjuvant: Post-surg., Post-surgery.

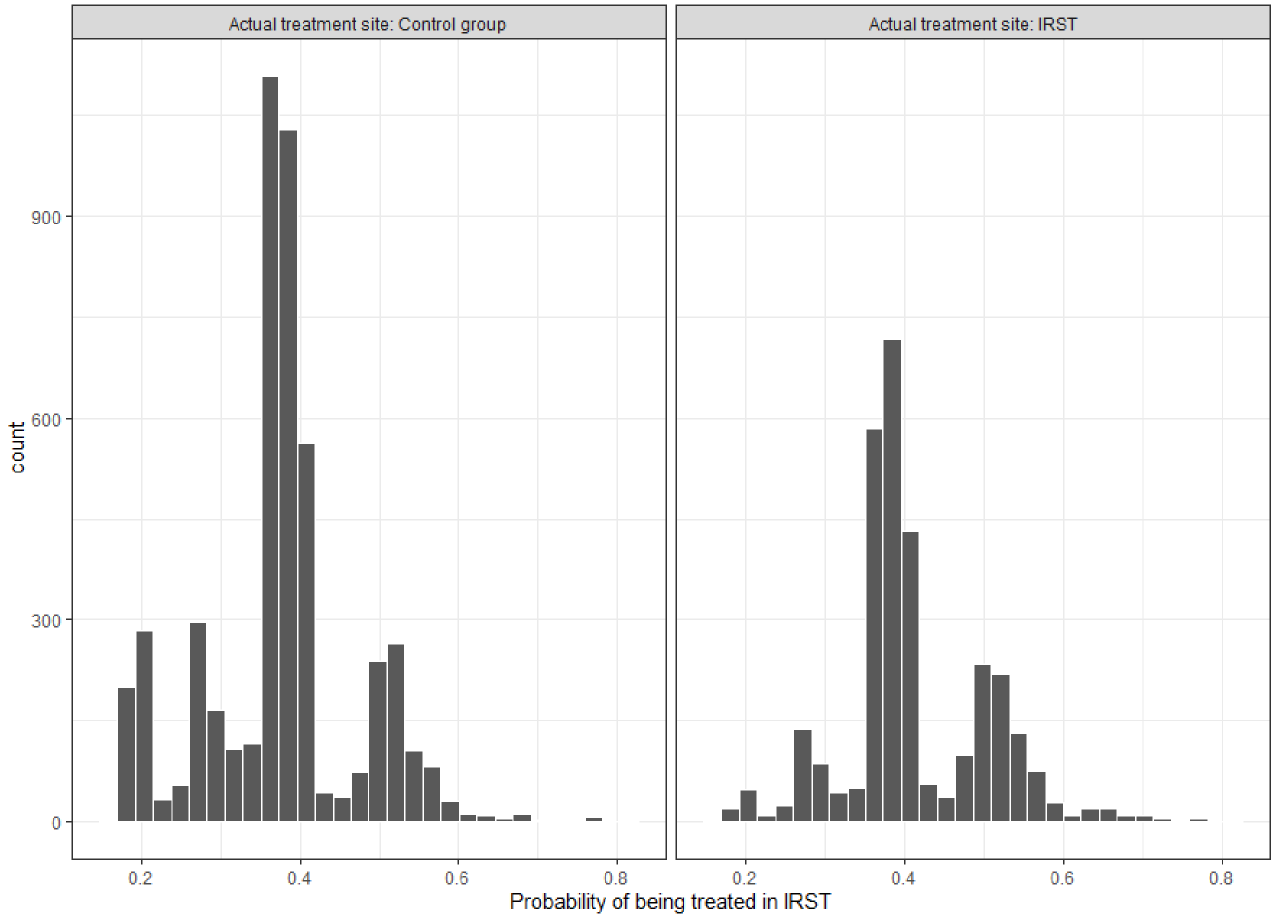

Figure S1. Common support requirement assessment. Propensity scores distribution of IRST and control groups and propensity scores distribution domain overlap assessment.

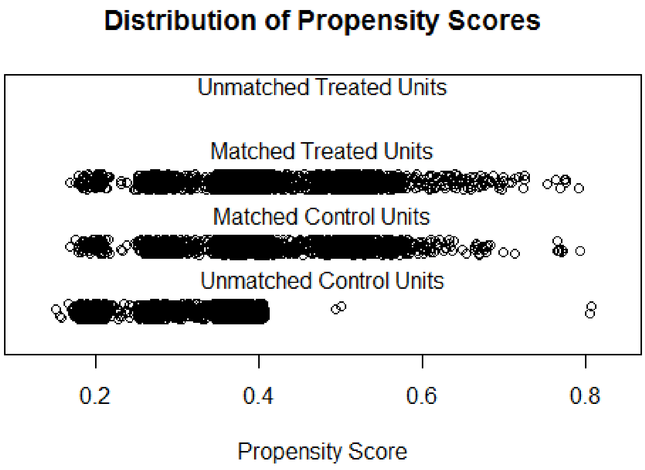

Note: Treated Units=IRST group; Control Units=Control group.

Figure S2: Distribution of propensity scores after the nearest neighbor matching. The matched treated units represent patients referred to IRST (N=3112), while the matched control units represent patients

(N=3112). The unmatched (i.e. the patients who had more distant characteristics) control units (N=1797) were excluded.

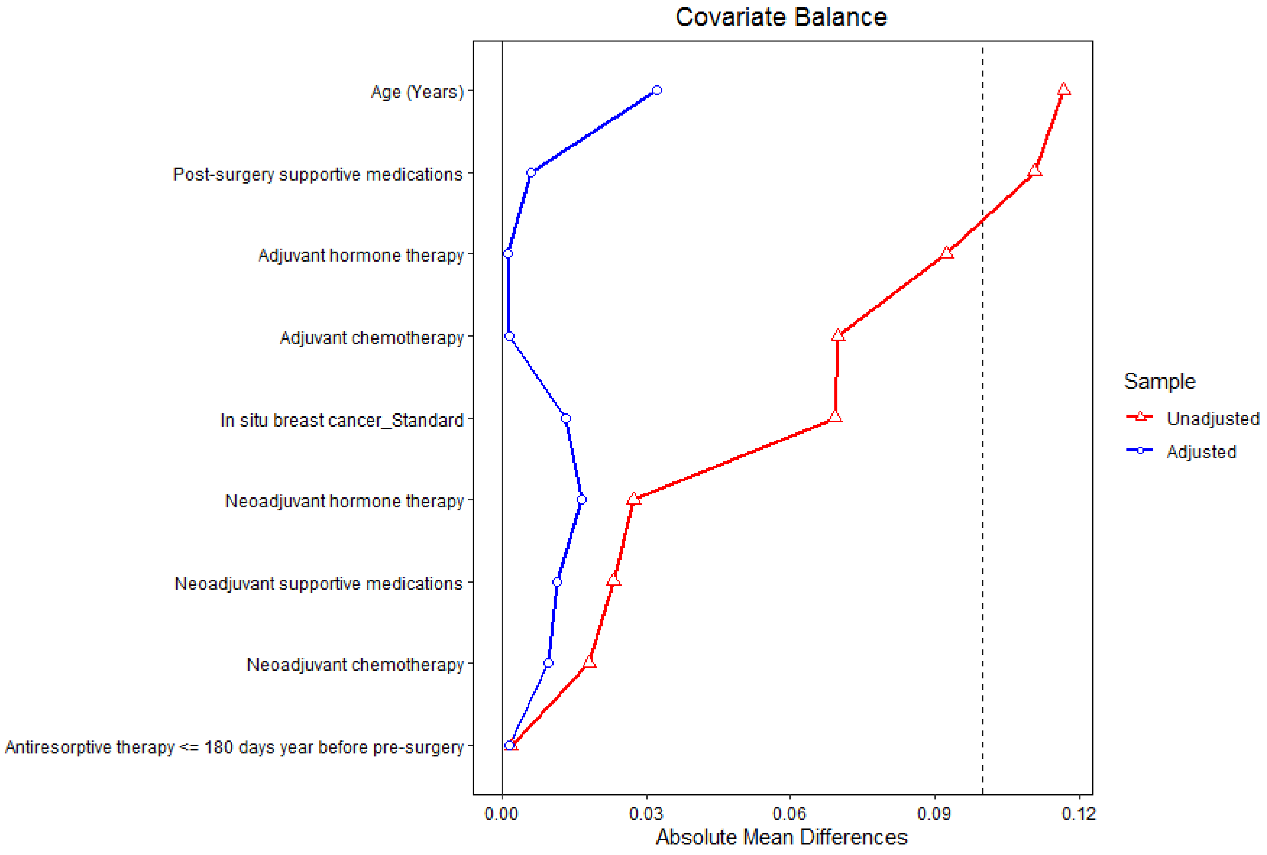

Figure S3: Love plot of absolute standardized mean differences for baseline covariates before (red) and after (blue) adjustment. The dashed vertical line indicates the 0.1 threshold for acceptable balance.
